# Total Calcium Intake Is Associated With Trabecular Bone Density in Adolescent Girls With Type 1 Diabetes

**DOI:** 10.1002/jbm4.10813

**Published:** 2023-09-19

**Authors:** Rylee K Saunders, Kathleen M Kilroe, Taïsha V. Joseph, Signe Caksa, Mary L Bouxsein, Madhusmita Misra, Deborah M Mitchell

**Affiliations:** ^1^ Endocrine Unit Massachusetts General Hospital and Harvard Medical School Boston MA USA; ^2^ Center for Advanced Orthopaedic Studies Beth Israel Deaconness Medical Center and Harvard Medical School Boston MA USA; ^3^ Division of Pediatric Endocrinology Massachusetts General Hospital and Harvard Medical School Boston MA USA; ^4^ Neuroendocrine Unit Massachusetts General Hospital and Harvard Medical School Boston MA USA

**Keywords:** BONE DENSITY, CALCIUM, PEDIATRICS, TYPE 1 DIABETES

## Abstract

Type 1 diabetes (T1D) confers an increased risk of fracture and is associated with lower bone mineral density (BMD) and altered microarchitecture compared with controls. Adequate calcium (Ca) intake promotes bone mineralization, thereby increasing BMD. The objective of this analysis was to evaluate the associations of total daily Ca intake with bone outcomes among youth with T1D. This was a cross‐sectional analysis of girls ages 10–16 years with (*n* = 62) and without (*n* = 60) T1D. We measured Ca intake with a validated food‐frequency questionnaire and BMD, microarchitecture, and strength estimates with dual‐energy X‐ray absorptiometry and high‐resolution peripheral quantitative computed tomography. Total daily Ca intake did not differ between groups (950 ± 488 in T1D versus 862 ± 461 mg/d in controls, *p* = 0.306). Serum 25OHD was lower in T1D (26.3 ± 7.6 versus 32.6 ± 9.0 ng/mL, *p* = <0.001), and parathyroid hormone (PTH) was higher in T1D (38.9 ± 11 versus 33.4 ± 9.7 pg/mL, *p* = 0.004). Trabecular volumetric BMD and thickness at the tibia were lower in T1D (*p* = 0.013, *p* = 0.030). Ca intake correlated with trabecular BMD at the radius and tibia among T1D participants (β = 0.27, *p* = 0.047, and β = 0.28, *p* = 0.027, β = 0.28, respectively) but not among controls (p_interaction_ = 0.009 at the radius, p_interaction_ = 0.010 at the tibia). Similarly, Ca intake was associated with estimated failure load at the tibia in T1D but not control participants (*p* = 0.038, β = 0.18; p_interaction_ = 0.051). We observed the expected negative association of Ca intake with parathyroid hormone in controls (*p* = 0.022, β = −0.29) but not in T1D participants (p_interaction_ = 0.022). Average glycemia as measured by hemoglobin A1c did not influence the relationship of Ca and PTH among participants with T1D (p_interaction_ = 0.138). These data suggest that youth with T1D may be particularly vulnerable to dietary Ca insufficiency. Increasing Ca intake may be an effective strategy to optimize bone health in this population. © 2023 The Authors. *JBMR Plus* published by Wiley Periodicals LLC. on behalf of American Society for Bone and Mineral Research.

## Graphical abstract

To better understand the pathophysiology of altered skeletal microarchitecture in youth with type 1 diabetes (T1D), we investigated the role of calcium intake in 122 girls ages 10–16 years, of whom 62 had T1D. We found that calcium intake was associated with trabecular bone mineral density at the distal radius and tibia and with estimated failure load at the tibia in girls with T1D, although not in the control participants. These data suggest that optimizing calcium intake may improve skeletal health in this population.

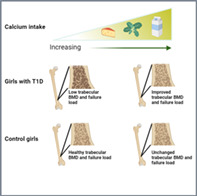

## Introduction

Fracture risk is increased in individuals with type 1 diabetes (T1D),^(^ ^1^, ^2^, ^3^ ^)^ and this excess risk begins during childhood.^(^ ^3^ ^)^ Multiple studies have shown low bone mineral density (BMD) and altered microarchitecture among youth with T1D.^(^ ^4^, ^5^, ^6^ ^)^ Childhood is an important time for bone development, as approximately 25% of adult bone mineral content (BMC) is accrued during the pubertal growth spurt,^(^ ^7^ ^)^ and bone microarchitecture also undergoes changes during puberty.^(^ ^8^, ^9^, ^10^ ^)^ Because T1D is typically diagnosed in childhood before the attainment of peak bone mass,^(^ ^11^ ^)^ many patients with T1D will experience its negative effects on bone health during this critical time. T1D may impair bone mass accrual and hinder geometry and microarchitecture development,^(^ ^12^ ^)^ potentially contributing to the increased risk of fracture across the life span.^(^ ^3^ ^)^

The underlying pathophysiology of bone fragility in T1D is not fully understood, and likely many factors contribute. One such factor may be inadequate calcium availability during bone accrual in childhood. Calcium contributes to bone strength via mineralization of the bone matrix,^(^ ^13^ ^)^ with calcium accretion peaking during adolescence.^(^ ^7^ ^)^ Interventional studies have demonstrated that increased calcium intake enhances BMD in peripubertal children as well as bone mass accrual in prepubertal girls,^(^ ^14^, ^15^ ^)^ although there is uncertainty about how long and to what extent this effect persists after discontinuation of the intervention.^(^ ^16^, ^17^ ^)^ Among youth with diabetes, most studies show no difference in total calcium intake compared with nondiabetic peers.^(^ ^18^, ^19^, ^20^ ^)^ In addition, few studies have found associations of calcium intake with bone health in T1D participants.^(^ ^21^ ^)^ However, excess urinary calcium loss in T1D may alter calcium homeostasis. In a study assessing calcium absorption and retention among adolescent girls with T1D, 25% of participants had negative calcium retention and elevated urinary calcium excretion.^(^ ^22^ ^)^ Although studies have shown positive associations of calcium intake with BMD and bone mineral content (BMC) in healthy children,^(^ ^23^, ^24^ ^)^ the effect of calcium intake on bone mass and microarchitecture in children with T1D remains uncertain.

In a cohort of girls ages 10–16 years with and without T1D, we have previously shown trabecular BMD, microarchitecture, and hip geometry are altered in T1D compared with controls.^(^ ^5^, ^12^ ^)^ The objective of this secondary analysis was to assess dietary and supplemental calcium intake in this cohort to investigate the association of calcium intake with BMD and microarchitecture. We hypothesized that higher average calcium intake would be associated with higher BMD and more robust microarchitecture among participants with T1D.

## Materials and Methods

### Study participants

Details of participants have been previously published.^(^ ^5^ ^)^ Girls aged 10–16 years with (*n* = 62) or without (*n* = 60) T1D were enrolled. Exclusion criteria included conditions known to impact bone health, including hyperthyroidism, celiac disease, hypogonadism, renal disease, vitamin D deficiency (25OHD < 20 ng/mL with a parathyroid hormone [PTH] concentration above the upper limit of normal), and underweight or obesity (body mass index [BMI] ≤5th or ≥95th percentile for age, respectively). All girls with T1D had a disease duration of at least 1 year and had positive autoantibodies or were diagnosed by their primary endocrinologist to have T1D based on clinical presentation. One subject from the original cohort was excluded because, on follow‐up visits, she was found to have metabolic bone disease. Informed consent and assent were obtained from a parent/guardian and the participants, respectively. This study was approved by the Partners Human Research Committee and was registered with ClinicalTrials.gov (ID NCT02140424).

### Clinical and biochemical investigation

Race was self‐identified by the subjects. Subjects had a standardized medical history and physical examination performed, including breast Tanner staging^(^ ^25^ ^)^ by a single pediatric endocrinologist (DMM) as well as height and metabolic weight measurements. Dietary and supplemental calcium intake were measured by a validated food‐frequency questionnaire.^(^ ^26^ ^)^ Medical records were reviewed for age and antibody status at T1D diagnosis. To determine bone age, a radiograph of the left hand was obtained and read by a single pediatric endocrinologist (DMM).^(^ ^27^ ^)^ Fasting blood samples were collected before 10:00 a.m.; HbA1c and PTH were measured in real time by a reference laboratory (LabCorp, Burlington, NC, USA), 25OHD was measured in real time by liquid chromatography‐mass spectrometry (Mayo Medical Laboratories, Rochester, MN, USA). Physical activity was measured by the Physical Activity Questionnaire for Adolescents (PAQ‐A).^(^ ^28^ ^)^

### DXA imaging

Scans of the whole body less head (subtotal body), spine, hip, and distal 1/3 radius were obtained (Hologic Horizon‐A, Marlborough, MA, USA) for BMD and body composition (least significant change 0.042, 0.024, 0.048, 0.024 g/cm^2^ for spine, total hip, femoral neck, and distal radius, respectively). *Z*‐scores were generated with Hologic Apex 3.3 software.

### HRpQCT imaging

We measured volumetric densities and microarchitecture of the distal radius (7% site) and distal tibia (8% site) with HRpQCT (XtremeCT, Scanco Medical AG, Bruttisellen, Switzerland).^(^ ^29^, ^30^ ^)^ The nondominant limb was scanned unless there was a history of fracture, in which case the dominant side was scanned. Fourteen radius scans were excluded (4 from T1D group and 10 from control group) from the analysis because of motion artifact. Microfinite element analysis (FEA) was used to estimate failure load.^(^ ^31^ ^)^

### Statistical analysis

Study data were collected and managed using the REDCap electronic data capture software (https://projectredcap.org/resources/citations) hosted at Partners Healthcare.^(^ ^32^ ^)^ Analyses were performed with R Studio 4.2.1 (R Foundation for Statistical Computing, Vienna, Austria) and Stata 12.0 (StataCorp LP, College Station, TX, USA). Statistical significance was defined as 2‐sided *p* < 0.05; as this was an exploratory analysis, we did not adjust for multiple comparisons. This is a secondary, cross‐sectional data analysis, and no relevant power calculations were performed. Demographic and clinical characteristics are reported as mean ± standard deviation for continuous data and as percentages for categorical data. Continuous data were compared by the Student's *t* test and proportions by Fisher's exact test. Dual‐energy X‐ray absorptiometry (DXA) data were compared by the Student's *t* test for unadjusted comparisons and by multivariable regression when adjusting for bone age, height, and weight. High‐resolution peripheral quantitative computed tomography (HRpQCT) data were compared using multivariable regression, adjusting for bone age, height, and weight. Interaction terms were tested to evaluate whether the associations of calcium intake with various parameters differed among subjects with or without T1D. Added variable plots were used to visualize the relationships between calcium intake and bone outcomes when adjusted for bone age, height, and weight.

## Results

### Subject characteristics

Table 1 shows the clinical characteristics of the 122 study participants; the groups were well‐matched for age, bone age, and age at menarche, although fewer of the T1D participants were premenarchal (*p* = 0.011). No participants with T1D had any diabetes‐related microvascular complications, including microalbuminuria, nephropathy, neuropathy, or retinopathy. As expected, HbA1c was higher among subjects with T1D than control subjects. The *Z*‐scores for BMI and weight were significantly higher among T1D subjects. Daily calcium and vitamin D intake were similar between groups, though subjects with T1D had lower 25OHD and higher PTH concentrations compared with controls (*p* < 0.001 and *p* = 0.004, respectively). Fasting urine calcium/creatinine ratio was lower in T1D (*p* = 0.043). Physical activity was similar between groups.

**Table 1:** Clinical Characteristics of Control Subjects and Subjects With Type 1 Diabetes

Variable	T1D	Control	
	(*n* = 62)	(*n* = 60)	*p* Value
Age (years)	13.6 ± 1.7	13.5 ± 1.9	0.782
Bone age (years)	13.9 ± 1.8	13.9 ± 2.2	0.995
Premenarche (*n*, %)	4 (6%)	14 (23%)	**0.011**
Age at menarche (years)	12.6 ± 1.1	12.2 ± 1.0	0.100
Age at onset (T1D) (years)	8.8 ± 3.0	—	—
Duration of disease (T1D) (years)	4.8 ± 3.2	—	—
Fracture history (*n*, %)	22 (35%)	26 (43%)	0.463
Weight (*Z*‐score)	0.7 ± 0.8	0.4 ± 0.9	**0.047**
Height (*Z*‐score)	0.3 ± 1.2	0.4 ± 0.9	0.422
BMI (*Z*‐score)	0.7 ± 0.7	0.3 ± 0.8	**0.003**
Physical activity score	2.2 ± 0.5	2.3 ± 0.5	0.067
Dietary calcium intake (mg/d)	899 ± 475	816 ± 452	0.326
Total calcium intake (mg/d)	950 ± 488	862 ± 461	0.306
Total vitamin D intake (IU/d)	198 ± 267	256 ± 467	0.404
Calcium (mg/dL)	9.6 ± 0.4	9.6 ± 0.3	0.944
TP/GFR (mg/dL)	3.8 ± 0.6	4.0 ± 0.6	0.208
HgbA1c (%)	8.6 ± 1.4	5.4 ± 0.3	**<0.0001**
PTH (pg/mL)	38.9 ± 11.0	33.4 ± 9.7	**0.004**
25‐OHD (ng/mL)	26.3 ± 7.6	32.6 ± 9.0	**<0.0001**
Fasting urine calcium/creatinine ratio	0.06 ± 0.06	0.08 ± 0.06	**0.043**
Phosphate (mg/dL)	4.2 ± 0.5	4.3 ± 0.6	0.409
Race (self‐described)			
White	53 (85%)	55 (92%)	0.399
Black	1 (2%)	2 (3%)	
Multiple/other	8 (13%)	3 (5%)	
Tanner stage (*n*)			
1	1 (2%)	4 (7%)	0.257
2	8 (12%)	12 (20%)	
3	11 (16%)	5 (8%)	
4	13 (19%)	9 (15%)	
5	29 (43%)	30 (50%)	

*Note*: Data presented as mean ± standard deviation or *n* (%). Comparisons evaluated by *t* test or Fisher's exact test. Of subjects who identified race as multiple/other, subjects with T1D identified as “Native American and Black” (*n* = 1), “Hispanic” (*n* = 5), “White and Pacific Islander” (*n* = 1), and “White, Black, and Native American” (*n* = 1). Control subjects identified as “Asian and Caucasian” (*n* = 1), “White and Black” (*n* = 1), “Mixed Brazilian and White” (*n* = 1).

Abbreviation: 25‐OHD = 25‐hydroxyvitamin D; BMI = body mass index; HgbA1c = hemoglobin A1c; PTH = parathyroid hormone; T1D = type 1 diabetes; TP/GFR = tubular PO_4_ reabsorption per dL/glomerular filtration rate.

### Areal BMD and HR‐pQCT volumetric BMD and microarchitecture

As previously shown,^(^ ^5^ ^)^ we found no significant differences in areal BMD (aBMD) between T1D and control subjects at the whole body less head, lumbar spine, total hip, femoral neck, and 1/3 radius sites both with univariable comparisons and after adjustment for bone age, height, and weight (Supplemental Table S1). At the distal radius, cortical volumetric BMD (vBMD) was significantly lower among the T1D cohort (*p* = 0.013). At the distal tibia, trabecular vBMD, trabecular thickness, and estimated failure load were significantly lower in the T1D participants (*p* = 0.013, *p* = 0.030, *p* = 0.039, respectively). Cortical porosity of the tibia was significantly higher in those with T1D (*p* = 0.046).

### Total calcium intake and skeletal parameters

**Fig. 1:**
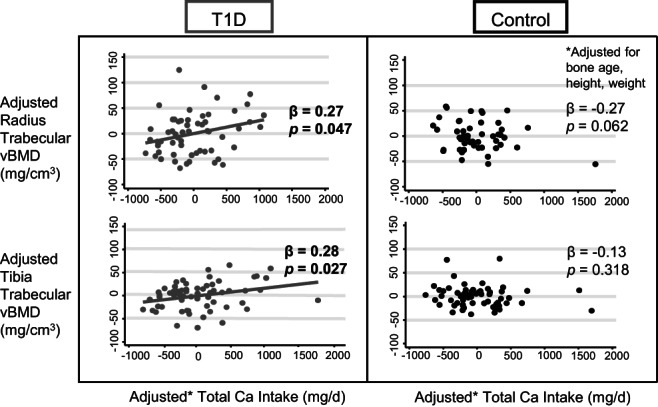
Partial regression plots for associations of calcium (Ca) intake with trabecular bone mineral density (BMD). Trabecular volumetric BMD (vBMD) and total calcium intake adjusted for bone age, height, and weight as covariates. Negative trabecular vBMD and total calcium intake values represent the residuals after adjustment.

As seen in Table 2, total calcium intake was not correlated with aBMD as measured by DXA at any site within the whole cohort nor after stratifying by T1D status. Volumetric imaging by HRpQCT revealed that, while calcium intake was not associated with trabecular BMD in the combined cohort nor among control participants, among T1D participants, higher calcium intake was associated with higher trabecular BMD (β = 0.27, *p* = 0.047 at the radius and β = 0.28, *p* = 0.027 at the tibia). Interaction analysis demonstrated a positive interaction of T1D and calcium intake on trabecular BMD such that higher calcium intake was associated with a greater increase in trabecular BMD among T1D compared with control participants (*p* = 0.009 at the radius and *p* = 0.010 at the tibia). Neither trabecular number nor thickness were significantly associated with calcium intake among T1D participants. Among control participants, we observed a negative correlation of calcium intake with trabecular thickness at the distal radius (β = −0.38, *p* = 0.004). Cortical parameters, including vBMD, thickness, and porosity, were not associated with calcium intake either in the whole cohort or in either group after stratification. Among T1D participants, failure load at the distal tibia was significantly associated with calcium intake (β = 0.18, *p* = 0.038) with a borderline significant interaction of T1D with calcium intake on tibia failure load (*p* = 0.051). Figure 1 provides visualization of the associations of calcium intake with trabecular vBMD after adjusting for bone age, height, and weight. We evaluated a potential role of 25OHD by adding this measure to our base model. We continued to observe a negative association of calcium intake with trabecular thickness at the distal radius in controls (β = −0.33, *p* = 0.013) and positive associations of calcium intake with trabecular vBMD and estimated failure load at the distal tibia in T1D participants (β = 0.28, *p* = 0.031 and β = 0.17, *p* = 0.043, respectively). However, the association of calcium intake with trabecular vBMD at the distal radius in T1D participants was no longer significant (β = 0.26, *p* = 0.057).

**Table 2:** Association of Total Calcium Intake With Bone Density and Microarchitecture

	Whole cohort (*n* = 122)			T1D (*n* = 62)		Control (*n* = 60)		
	Parameter	β	*p* Value	β	*p* Value	β	*p* Value	
	WBLH aBMD	0.05	0.347	0.14	0.118	−0.01	0.848	
	Spine aBMD	0.01	0.828	0.07	0.391	−0.02	0.817	
DXA	TH aBMD	0.06	0.333	0.15	0.151	−0.01	0.867	
	FN aBMD	0.07	0.282	0.17	0.102	0.00	0.964	
	Radius aBMD	0.02	0.737	−0.01	0.874	0.04	0.322	
	Total area	0.09	0.298	0.06	0.566	0.13	0.332	
	Cort Th	−0.01	0.980	−0.01	0.855	0.04	0.679	
	Trab vBMD	0.07	0.459	0.27	**0.047**	−0.27	0.062	
	Trab N	0.13	0.181	0.22	0.099	0.02	0.868	
Radius HRpQCT	Trab Th	−0.04	0.710	0.20	0.141	−0.38	**0.004**	
	Trab Sep	−0.11	0.279	−0.21	0.109	0.03	0.822	
	Cort vBMD	−0.02	0.770	−0.06	0.440	0.06	0.490	
	Cort Po	0.04	0.655	0.12	0.286	−0.09	0.467	
	Failure load	0.07	0.263	0.09	0.294	0.05	0.522	
	Total area	0.07	0.380	0.05	0.653	0.11	0.327	
	Cort Th	0.05	0.381	0.08	0.350	0.03	0.713	
	Trab vBMD	0.08	0.387	0.28	**0.027**	−0.13	0.318	
	Trab N	0.03	0.707	0.15	0.215	−0.10	0.434	
Tibia HRpQCT	Trab Th	0.05	0.656	0.17	0.175	−0.04	0.752	
	Trab Sep	−0.05	0.531	−0.14	0.248	0.08	0.528	
	Cort vBMD	−0.04	0.401	−0.08	0.178	0.04	0.544	
	Cort Po	0.07	0.307	0.12	0.201	−0.06	0.507	
	Failure load	0.06	0.225	0.18	**0.038**	0.00	0.989	

*Note*: Comparisons evaluated by linear regression adjusting for bone age, height, and weight. Data presented as *p* value and standardized beta coefficient.

Abbreviation: aBMD = areal bone mineral density; Cort = cortical; DXA = dual‐energy X‐ray absorptiometry; FN = femoral neck; HRpQCT= high‐resolution peripheral quantitative computed tomography; N = number; Po = porosity; Sep = separation; T1D = type 1 diabetes; Th = thickness; TH = total hip; Trab = trabecular; vBMD = volumetric bone mineral density; WBLH = whole body less head.

### The relationship of parathyroid hormone with calcium intake and bone

**Fig. 2:**
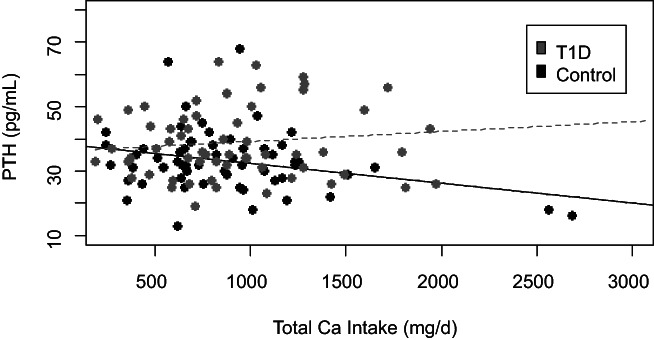
The relationship of total calcium (Ca) intake and parathyroid hormone (PTH) in type 1 diabetes (T1D) and control subjects as graphed by linear regression. The solid line indicates a significant interaction of diabetes with calcium intake on the suppression of PTH (*p*_interaction_ = 0.022, β =0.24), with a negative association of calcium intake with PTH in controls (*p* = 0.022, β = −0.29). The dashed line indicates no significant association of calcium intake with PTH in T1D (*p* = 0.370).

As seen in Figure 2, there was no overall association of PTH and calcium intake among the whole cohort (*p* = 0.691). As expected, control participants showed an inverse association of calcium intake with PTH, such that increased calcium intake was associated with lower circulating PTH (*p* = 0.022, β = −0.29). However, among T1D participants, we did not observe the expected suppression of PTH with increasing calcium intake (*p* = 0.370), and we observed a significant interaction of diabetes with calcium intake on the suppression of PTH (*p*_interaction_ = 0.022, β = 0.24). After adding 25OHD to our base model, the results were similar. Specifically, we did not observe PTH suppression with increasing calcium intake in T1D participants (*p* = 0.248) but continued to observe a significant negative association of calcium intake with PTH in controls (*p* = 0.025, β = −0.29). The interaction of diabetes with calcium intake on the suppression of PTH remained significant (*p* = 0.017, β = 0.53).

### The relationship of glycemic control with calcium and bone parameters

After stratifying by diabetes status, we did not observe significant associations of HbA1c with dietary calcium intake (*p* = 0.623 in control and *p* = 0.606 in T1D) nor with serum calcium (*p* = 0.263 in control and *p* = 0.344 in T1D). We did observe a significant positive association of HbA1c with urinary calcium to creatinine ratio in T1D (*p* = 0.046) but not in control participants (*p* = 0.287). As previously shown,^(^ ^5^ ^)^ HbA1c was negatively associated with trabecular thickness at the tibia (*p* = 0.049) but no other bone density or microarchitecture parameter.

To test the hypothesis that polyuria secondary to hyperglycemia leads to excess urinary calcium excretion, we investigated whether average glycemia modulated the effect of calcium intake on PTH in those with T1D by stratifying at the median HbA1c value (T1D Low = A1c <8.5%, T1D high = A1c ≥ 8.5%; *n* = 29 versus *n* = 32, respectively). We did not find significant associations of calcium intake with PTH in the T1D Low or T1D High groups (*p* = 0.827, *p* = 0.173, respectively), and there was no interaction of calcium intake with HbA1c on PTH (*p*_interaction_ = 0.138). Additionally, we did not find significant interactions of HbA1c and calcium intake on trabecular vBMD at either the radius or tibia.

## Discussion

The prevention of fracture later in life starts during childhood and adolescence by maximizing peak bone mass and optimizing bone geometry and microarchitecture. As T1D alters bone microarchitecture in youth,^(^ ^4^, ^5^, ^6^ ^)^ the goal of this analysis was to determine to what extent dietary calcium intake impacts volumetric BMD and microarchitecture in adolescent girls with T1D. As in most prior studies, the mean total daily calcium intake did not differ between T1D and healthy participants.^(^ ^18^, ^20^ ^)^ Our data demonstrate an association of calcium intake with trabecular BMD at the distal radius and tibia and estimated bone strength at the tibia in girls with T1D, consistent with many prior studies.^(^ ^7^, ^14^ ^)^ This relationship, however, was not observed in healthy control participants.

Our data suggest that optimizing calcium intake may benefit bone mineralization in youth with T1D. In a randomized controlled trial assessing bone accrual with calcium fortified foods, healthy adolescent girls receiving calcium supplementation displayed a higher percentage of bone accrual than those without, with supplementation displaying a greater benefit in those who had a calcium intake <850 mg/d, and this difference persisted for over 3 years post intervention.^(^ ^15^, ^33^ ^)^ Although many but not all studies of calcium supplementation have shown BMC and BMD improvements in healthy adolescents,^(^ ^23^, ^24^, ^34^ ^)^ several prior studies investigating the relationship of calcium intake with BMD in T1D patients have not shown significant associations. Previous studies assessing calcium intake, whether dietary or supplemental, have primarily utilized DXA and pQCT to assess skeletal outcomes.^(^ ^18^, ^19^, ^20^ ^)^ This article is the first to utilize HRpQCT technology to evaluate the association of calcium intake with compartmental BMD and microarchitecture in youth with T1D. HRpQCT imaging enables more precise assessment of BMD of cortical and trabecular bone as well as evaluation of microarchitecture. Importantly, HRpQCT‐derived measures of compartmental BMD and microarchitecture are predictive of fracture risk in adults even after adjusting for areal BMD.^(^ ^35^ ^)^ Our data are consistent with a murine model investigating the relationship of calcium intake and bone microarchitecture in which calcium deficiency led to low trabecular volumetric BMD and altered bone microarchitecture.^(^ ^36^ ^)^ The association of calcium intake with indices of bone strength in our cohort suggests that inadequate calcium availability may contribute to impaired acquisition of bone during childhood and adolescence, leading to the increased fracture risk among people with T1D.

The period of adolescence requires high rates of calcium accretion to mineralize newly generated bone, and this can be achieved by an increased retention of dietary calcium.^(^ ^7^ ^)^ Studies have previously measured calcium retention and absorption in healthy girls aged 9–15 years and observed positive retention throughout adolescence.^(^ ^37^, ^38^ ^)^ Weber and colleagues measured retention and absorption in a cohort of girls with T1D aged 9–16 years.^(^ ^22^ ^)^ This cohort had similar calcium intake, mean percent calcium absorption, and true calcium absorption compared with the nondiabetic cohort, yet the T1D participants had significantly greater urine calcium excretion. Excess calcium excretion in the setting of hyperglycemia‐induced osmotic diuresis may underlie the observed differences in the association of calcium intake with vBMD between girls with and without T1D in our cohort, as well as the differences in PTH suppression with increasing calcium intake. Of note, in our cohort, the mean fasting urine calcium/creatinine ratio was significantly lower in the T1D participants compared with controls, which seems to contradict this hypothesis. However, we do not have measures of urine calcium excretion in the post‐prandial state, which may better reflect this expected effect. This may drive a higher calcium requirement in girls with T1D, and optimization of calcium intake in youth with diabetes could strengthen the beneficial qualities of dietary calcium on bone health. However, excessive calcium supplementation may result in hypercalciuria and other complications. The lack of PTH suppression with higher calcium intake may alternatively be due to insufficient calcium absorption. Specifically, lower 25OHD and an altered gastrointestinal microbiome in T1D may also contribute to an impaired effect of calcium intake by attenuating calcium absorption in the gut, although, of note, our findings were largely similar after adjusting for circulating 25OHD.^(^ ^39^, ^40^ ^)^

Calcium is a threshold nutrient, such that calcium intake is adequate when the need for bone mineralization is met and calcium retention maximized.^(^ ^41^ ^)^ In youth ages 9–18 years, the recommended daily allowance (RDA) of calcium is 1300 mg/d.^(^ ^42^ ^)^ However, data regarding the calcium threshold are conflicting, with estimates ranging from 970 mg/d to 2 g/d.^(^ ^43^, ^44^ ^)^ Within our cohort, both those with T1D and those without had a mean daily calcium intake less than the RDA, with only 13% of the T1D group and 8% of the control group meeting the RDA. Our results show that the control participants did not have an association of calcium intake and BMD, and this may be due to the control participants meeting their threshold.

We also observed a significant negative correlation between calcium intake and trabecular thickness at the radius in our control group. This finding is unexplained. Although it may reflect genuine physiology, prior interventional studies of calcium intake in healthy children suggest that increased calcium intake improves trabecular BMC,^(^ ^45^, ^46^ ^)^ suggesting that this finding may be due to type I error.

A strength of our investigation is that this is a relatively large and well‐phenotyped cohort of adolescents with T1D who have undergone areal and volumetric bone imaging. Limitations include that our cohort includes only girls with a narrow age range. Given that calcium accretion rates differ between boys and girls and across the stages of puberty,^(^ ^7^ ^)^ further investigation of the relationships of calcium intake with skeletal health in boys with T1D and in younger and older youth is critical. We did not obtain 24‐hour urine samples or stool samples, so we were unable to evaluate the effects of T1D on calcium absorption and excretion. This is an observational study, and our findings may be confounded by other health factors. Additionally, calcium intake was measured by a food‐frequency questionnaire and there may be imprecision of dietary recall. Because we did not adjust for multiple comparisons, it is possible that the significant differences we observed are due to chance.^(^ ^47^ ^)^

To summarize, our analysis demonstrates that among girls relatively early in their course of T1D, higher calcium intake is associated with greater trabecular BMD and estimated bone strength. Although our data show lower fasting urine calcium in T1D participants, the lack of PTH suppression with higher calcium intake suggests that urinary calcium losses in the setting of hyperglycemia‐induced diuresis may interfere with calcium retention, leading to a higher calcium requirement than nondiabetic peers. These data suggest that increasing calcium intake during childhood and adolescence may be an effective strategy to improve BMD and decrease fracture risk in T1D patients. Further studies more fully characterizing calcium homeostasis in T1D as well as interventional studies of calcium supplementation and effects on bone accrual will help to clarify the role of childhood calcium intake in diabetes‐associated skeletal fragility.

## Author Contributions

**Rylee K Saunders:** Conceptualization; formal analysis; writing – original draft. **Kathleen M Kilroe:** Data curation; investigation. **Taïsha V. Joseph:** Data curation; investigation. **Signe Caksa:** Data curation; investigation. **Mary L Bouxsein:** Investigation; writing – review and editing. **Madhusmita Misra:** Conceptualization; investigation; writing – review and editing. **Deborah M Mitchell:** Conceptualization; data curation; formal analysis; funding acquisition; investigation; methodology; supervision; writing – review and editing.

### Peer Review

The peer review history for this article is available at https://www.webofscience.com/api/gateway/wos/peer‐review/10.1002/jbm4.10813.

## Disclosures

None of the authors have any disclosures.

## Supporting information


**Data S1** Supplementary Information

## Data Availability

The data that support the findings of this study are available from the corresponding author upon request.
