# Supplementary material for: Total Calcium Intake Is Associated With Trabecular Bone Density in Adolescent Girls With Type 1 Diabetes
Source: JBMR Plus. 2023 Sep 19;7(11):e10813. doi: 10.1002/jbm4.10813 (PMC10652183; doi:10.1002/jbm4.10813)
Supplement: Supplementary file 1 — Data S1 Supplementary Information [file JBM4-7-e10813-s001.docx]

**Supplemental Table 1. Comparison of Areal Bone Density, Volumetric Bone Density, and Microarchitecture**

|  | **Parameter** | **T1D** | **Control** | ***P*** | ***P^a^*** |
| --- | --- | --- | --- | --- | --- |
|  | WBLH aBMD (g/cm^2^) | 0.837 ± 0.08 | 0.841 ± 0.09 | 0.862 | 0.352 |
|  | Spine aBMD (g/cm^2^) | 0.854 ± 0.14 | 0.847 ± 0.15 | 0.783 | 0.616 |
| **DXA** | TH aBMD (g/cm^2^) | 0.889 ± 0.13 | 0.887 ± 0.13 | 0.956 | 0.552 |
|  | FN aBMD (g/cm^2^) | 0.782 ± 0.12 | 0.794 ± 0.13 | 0.631 | 0.169 |
|  | Radius aBMD (g/cm^2^) | 0.613 ± 0.06 | 0.613 ± 0.07 | 0.976 | 0.162 |
|  | Total Area (mm^2^) | 164.2 ± 35.2 | 159.6 ± 34.3 | 0.487 | 0.609 |
|  | Cort Th (mm) | 0.977 ± 0.26 | 1.04 ± 0.28 | 0.214 | 0.053* |
|  | Trab vBMD (mgHA/cm^3^) | 149.3 ± 41.7 | 159.3 ± 28.6 | 0.142 | 0.179 |
|  | Trab N (1/mm) | 1.74 ± 0.32 | 1.78 ± 0.25 | 0.505 | 0.315 |
| **Radius XCT** | Trab Th (mm)  Trab Sep (mm) | 0.071 ± 0.01  0.526 ± 0.12 | 0.075 ± 0.01  0.500 ± 0.09 | 0.063  0.206 | 0.160  0.121 |
|  | Cort vBMD (mgHA/cm^3^) | 841.0 ± 76.7 | 867.3 ± 80.8 | 0.078 | **0.013** |
|  | Cort Po (%) | 2.57 ± 1.73 | 2.02 ± 1.73 | 0.103 | 0.099 |
|  | Failure Load (N) | 3401.5 ± 836 | 3563.5 ± 941 | 0.353 | 0.097 |
|  | Total Area (mm^2^) | 531.0 ± 89.9 | 517.6 ± 89.5 | 0.412 | 0.766 |
|  | Cort Th (mm) | 1.213 ± 0.3 | 1.231 ± 0.34 | 0.749 | 0.456 |
|  | Trab vBMD (mgHA/cm^3^) | 163.8 ± 30.3 | 175.6 ± 25.2 | **0.020** | **0.013** |
|  | Trab N (1/mm) | 1.80 ± 0.29 | 1.78 ± 0.29 | 0.703 | 0.398 |
| **Tibia XCT** | Trab Th (mm)  Trab Sep (mm) | 0.077 ± 0.01  0.498 ± 0.12 | 0.083 ± 0.01  0.493 ± 0.08 | **0.002**  0.766 | **0.030**  0.170 |
|  | Cort vBMD (mgHA/cm^3^) | 858.0 ± 85.7 | 865.8 ± 79.0 | 0.604 | 0.059 |
|  | Cort Po (%) | 3.77 ± 2.8 | 3.26 ± 2.05 | 0.257 | **0.046** |
|  | Failure Load (N) | 9056.5 ± 1831 | 9294.9 ± 2090 | 0.505 | **0.039** |

Data presented as mean ± standard deviation. Comparisons evaluated by t-test for univariate comparisons and by linear regression adjusting for bone age and height. Abbreviations: aBMD, areal bone mineral density; vBMD, volumetric bone mineral density; T1D, type 1 diabetes; WBLH, whole body less head; TH, total hip; FN, femoral neck; Cort, cortical; Trab; trabecular; Th, thickness; Sep, separation; Po, porosity; N, number. **^a^** Adjusted for bone age, height, and weight. Note that data in this table was previously published in Mitchell, JCEM 2020.
